# Supplementary material for: Combined Therapy Using Human Corneal Stromal Stem Cells and Quiescent Keratocytes to Prevent Corneal Scarring after Injury
Source: Int J Mol Sci. 2022 Jun 23;23(13):6980. doi: 10.3390/ijms23136980 (PMC9267074; doi:10.3390/ijms23136980)
Supplement: Supplementary file 1 [file ijms-23-06980-s001.zip › ijms-1765376-supplementary.pdf]

Combined therapy using human corneal stromal stem cells and quiescent keratocytes to prevent corneal scarring after injury

Vishal Jhanji, Mithun Santra, Andri K Riau, Moira L. Geary, Tianbing Yang, Elizabeth Rubin, Nur Zahirah Binte M Yusoff , Deepinder K Dhaliwal, Jodhbir S Mehta, Gary Hin-Fai Yam

Supplementary Materials

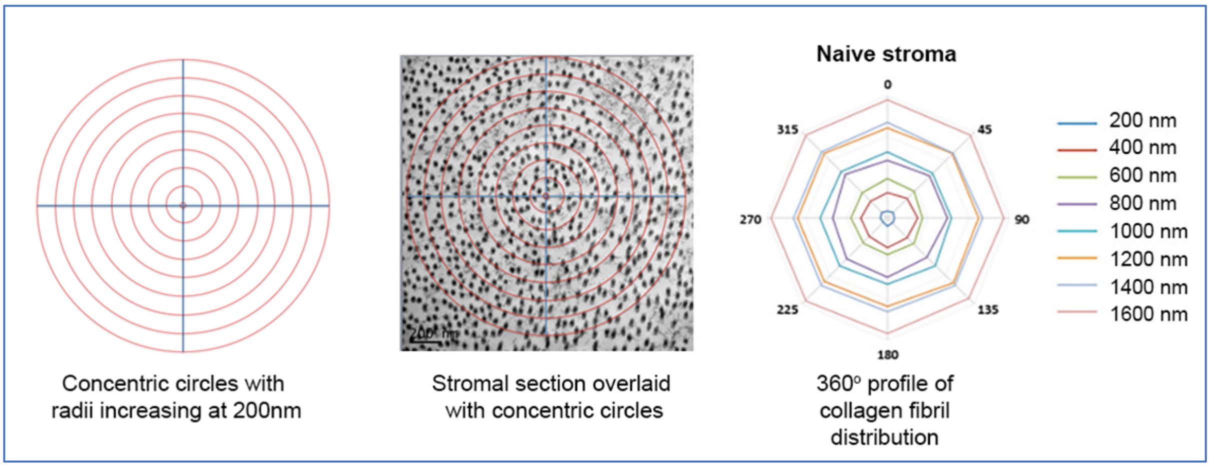

Supplementary Figure S1. 360° fibril distribution profile measured by concentric circle analysis method**.**

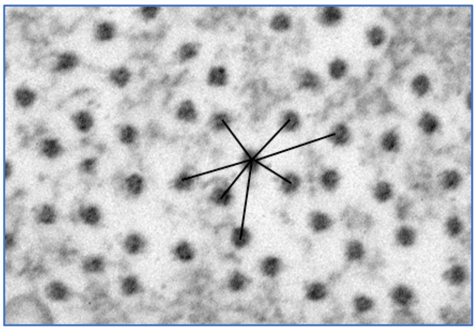

Supplementary Figure S2. Measurement of inter-fibrillar distances with a randomly chosen collagen fibril.

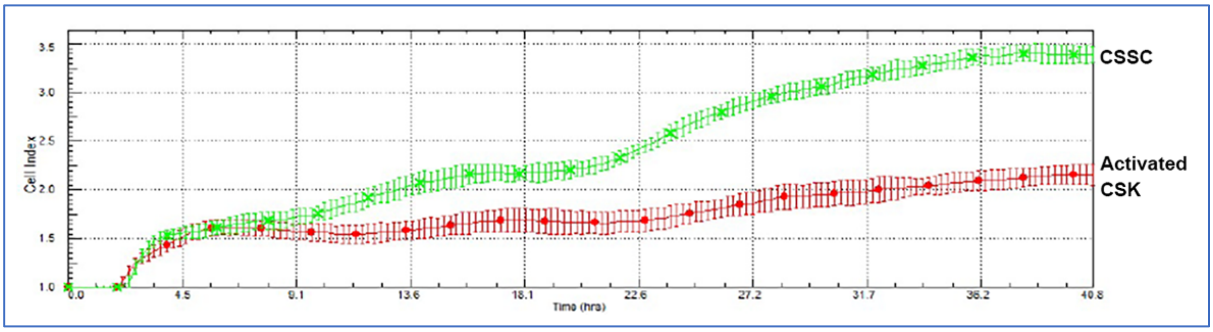

Supplementary Figure S3. Growth character of primary CSSC versus activated CSK from same donor cornea by xCelligence assay.

Supplementary Table S1. Donor cornea information

Lab no.	Age	Gender	Cause of Death	Hours from death to Optisol	Days in Optisol preservation
**436**	33	M	Multiple cerebral infarcts	5.5	7
**439**	57	F	Anoxia brain injury	6	9
**466**	51	F	Myocardial infarct	6	8
**515**	67	M	Atherosclerotic cardiovascular disease	5.5	6
**534**	25	M	Head trauma	6.5	9
**544**	41	M	STEMI heart attack	7.5	8
**555**	72	F	Decompensated heart failure	6.5	12
**572**	21	M	Anoxia brain injury	9	10
**576**	68	M	Myocardial infarct	10	9
**577**	65	M	Myocardial infarct	8.5	10
**621**	58	F	Acute myocardial infarct	8	8
**624**	65	M	Hypoxic ischemic encephalopathy	9.5	10
**641**	45	M	Gun shot	11	12

Supplementary Table S2. qPCR primers

	GenBank #	Forward (5'-3')	Reverse (5'-3')
Mouse ACP5	NM_013556.1	GTTGGATACAGGCCAGACTTTGTTG	GATTCAACTTGCGCTCATCTTAGGC
Mouse αSMA	NM_009696.3	TGTGCTGGACTCTGGAGATG	GAAGGAATAGCCACGCTCAG
Mouse Col3A1	NM_009930.2	CGTAAGCACTGGTGGACAGA	CGGCTGGAAAGAAGTCTGAG
Mouse CTSK	X94444.1	ATGTGGGGGCTCAAGGTTCTG	CATATGGGAAAGCATCTTCAGAGTC
Mouse FN1	NM010233.2	TACTCGAGCCCTGAGGATGG	GCAAGGCAACCACACTGACT
Mouse MMP9	NM_013599.5	CTTCTGGCGTGTGAGTTTCCA	ACTGCACGGTTGAAGCAAAGA
Mouse TNC	NM_035737.2	GACTGCCCTGGGAACTGTAA	CATAGCCTTCGAAGCACACA
Mouse TSG-6	NM_007115.3	AAGCACGGTCTGGCAAATACAAGC	ATCCATCCAGCAGCACAGACATGA
Human ABCG2	NM_004827	TGCAACATGTACTGGCGAAGA	TCTTCCACAAGCCCCAGG
Human ALDH3A1	NM_001135168	CATTGGCACCTGGAACTACC	GGCTTGAGGACCACTGAGTT
Human AQP1	NM_198098.2	AATACCCGGTGGGGAACAAC	CACACCATCAGCCAGGTCAT
Human αSMA	NM_001613.2	CTGTTCCAGCCATCCTTCAT	CCGTGATCTCCTTCTGCATT
Human B3GnT7	NM_145236.2	AGTCTCACCCCTGGTCAGTT	AGCAGTTAGTGGTGGTCACG
Human CD34	NM_001025109	CCTCAGTGTCTACTGCTGGTCT	GGAATAGCTCTGGTGGCTTGCA
Human CHST6	NM_021615.4	TACCGGCCTGTGTACTCTGA	ACTAATTTCGGGGGTGCGAG
Human Col3A1	NM_000090.3	TCTTTGAATCCTAGCCCATCTG	TGTGACAAAAGCAGCCCCATAA
Human CXCR4	NM_003467	CTCCTCTTTGTCATCACGCTTCC	GGATGAGGACACTGCTGTAGAT
Human FN1	NM_212482.4	CCACCCCCATAAGGCATAGG	GTAGGGGTCAAAGCACGAGTCATC
Human Kera	NM_007035	ATCTGCAGCACCTTCACCTT	CATTGGAATTGGTGGTTTGA
Human Lum	NM_002345.3	CCTGGTTGAGCTGGATCTGT	TGGTTTCTGAGATGCGATTG
Human MCP1	NM_002982.4	GAGAGGCTGAGACTAACCCAGA	ATCACAGCTTCTTTGGGACACT
Human Nestin	NM_006617	GCTCAGGTCCTGGAAGGTC	TAAGAAAGGCTGGCACAGGT
Human Pax6	NM_001604	CAATCAAAACGTGTCCAACG	TAGCCAGGTTGCGAAGAACT
Human SPARC	NM_003118.4	ATCTAAATCCACTCCTTCCACAG	CACCGTTAATGTATTCACTTAAATC

Supplementary Table S3. Antibodies used for immunostaining

Antibody anti-mouse epitope [clone]	Source
αSMA [1A4]	Invitrogen MA5-11547
Col3A1 [C-15]	Santa Cruz sc-8781
FN	Millipore AB2033
TNC [F-17]	Santa Cruz sc-9872

Antibody anti-human epitope [clone]	Source
ALDH3A1	Proteintech 15578-1-AP
αSMA [1A4]	Invitrogen MA5-11547
Col3A1 [C-15]	Santa Cruz sc-8781
FN	Millipore AB2033
Keratocan	Sigma HPA039321
Ki67	DAKO M7240
Lum	Sigma HPA001522
TNC [F-17]	Santa Cruz sc-9872
